# Pre-pulse inhibition and antisaccade performance indicate impaired attention modulation of cognitive inhibition in 22q11.2 deletion syndrome (22q11DS)

**DOI:** 10.1186/1866-1955-6-38

**Published:** 2014-09-19

**Authors:** Kathryn Louise McCabe, Rebbekah Josephine Atkinson, Gavin Cooper, Jessica Lauren Melville, Jill Harris, Ulrich Schall, Carmel Maree Loughland, Renate Thienel, Linda Elisabet Campbell

**Affiliations:** Schizophrenia Research Institute, Darlinghurst, Australia; Brain & Mind Research Institute, University of Sydney, Sydney, Australia; Centre for Translational Neuroscience & Mental Health, University of Newcastle, Callaghan, Newcastle Australia; School of Medicine & Public Health, University of Newcastle, Callaghan, Newcastle, Australia; School of Psychology, University of Newcastle, Science Offices, Callaghan, Ourimbah, NSW 2258 Australia; Minerals Industry Safety and Health Centre, University of Queensland, Brisbane, Australia

**Keywords:** Startle modification, PPI, PPF, Antisaccade, Neurocognition

## Abstract

**Background:**

22q11.2 deletion syndrome (22q11DS) is associated with a number of physical anomalies and neuropsychological deficits including impairments in executive and sensorimotor function. It is estimated that 25% of children with 22q11DS will develop schizophrenia and other psychotic disorders later in life. Evidence of genetic transmission of information processing deficits in schizophrenia suggests performance in 22q11DS individuals will enhance understanding of the neurobiological and genetic substrates associated with information processing. In this report, we examine information processing in 22q11DS using measures of startle eyeblink modification and antisaccade inhibition to explore similarities with schizophrenia and associations with neurocognitive performance.

**Methods:**

Startle modification (passive and active tasks; 120- and 480-ms pre-pulse intervals) and antisaccade inhibition were measured in 25 individuals with genetically confirmed 22q11DS and 30 healthy control subjects.

**Results:**

Individuals with 22q11DS exhibited increased antisaccade error as well as some evidence (trend-level effect) of impaired sensorimotor gating during the active condition, suggesting a dysfunction in controlled attentional processing, rather than a pre-attentive dysfunction using this paradigm.

**Conclusions:**

The findings from the present study show similarities with previous studies in clinical populations associated with 22q11DS such as schizophrenia that may indicate shared dysfunction of inhibition pathways in these groups.

## Background

22q11.2 deletion syndrome (22q11DS) is caused by a deletion on the long arm of chromosome 22 and is thought to affect between 30 and 60 genes associated with brain development and function [1]. Our ability to function and thrive in life is reliant on our capacity to select and attend to salient information in our environment and to ignore or inhibit non-salient information. The impairment of inhibitory information processes is associated with a wide range of psychiatric illnesses [2], including those common to 22q11DS such as schizophrenia. People with 22q11DS are at a greater risk of developing schizophrenia and other psychotic disorders [3–5], and genome-wide association studies (GWAS) indicate that the 22q11.2 deletion represents the strongest known genetic association in schizophrenia [1, 6]. Compared to control participants, people with schizophrenia as well as their first-degree relatives show poorer performance on inhibitory information processing measures of pre-pulse inhibition [PPI; [7–10]] and antisaccade inhibition [11, 12]. There is also evidence to suggest that pre-pulse inhibition is similarly impaired in 22q11DS [13, 14]. Inhibitory dysfunction observed prior to the onset of schizophrenia in groups at a genetic high risk for the disorder suggests that inhibitory dysfunction may represent a trait marker for developing schizophrenia [10]. Moreover, information processing difficulties may also account for some of the higher-order cognitive impairments that are commonly reported in 22q11DS such as executive dysfunction [15].

PPI is a measure of reduction of the acoustic startle response when a weak non-startling stimulus (pre-pulse; S2) is presented before a startling stimulus (S1) [16] and reflects pre-attentive and automatic sensorimotor gating mechanisms [17]. Reduced PPI has been consistently reported in people with schizophrenia [7, 9, 18], and it is considered a potential endophenotype for the disorder [9]. In healthy controls, typical levels of PPI are between 50% and 60% (though levels are influenced by factors such as stimulus type (e.g. white noise/pure tone) and interstimulus interval (ISI)) [18]. However, when variations of this paradigm include both attended and unattended conditions, individuals with schizophrenia show deficits in PPI *only* when instructed to *attend* to the pre-pulse [19, 20]. While reduced PPI is reported using unattended measures of PPI alone, it is not known whether impaired attention modulation of PPI is similarly observed in 22q11DS as in schizophrenia.

The antisaccade paradigm is another measure of inhibitory information processing and requires the participant to suppress a saccade to a stimulus (prosaccade) and generate a saccade to the mirror position (antisaccade) [21]. It is a voluntary executive control process regulated strongly by prefrontal cortical areas; however, both early pre-attentional or automatic and later controlled attentional processes contribute to generating the antisaccade [22]. In healthy controls, antisaccade error rate is estimated at 20% [23], and in schizophrenia, antisaccade error rate is significantly and consistently higher and is associated with poor executive functioning [24–26]. Despite evidence of poor executive functioning in 22q11DS [15, 27] and evidence linking antisaccade impairment to a locus on chromosome 22q11-12 [28], antisaccade performance has not been examined in 22q11DS.

The present study sought to extend previous inhibitory information processing studies in 22q11DS by examining attended and unattended measures of PPI, antisaccade inhibition and associations with executive functioning prior to the onset of overt psychosis. Consistent with previous studies [14], we expect to report reduced unattended PPI in participants with 22q11DS. Given evidence of attention deficits in 22q11DS, it is expected that when instructed to attend to the pre-pulse, compared to typically developing control participants, the 22q11DS group will fail to show normal attentional modulation of the startle response. In addition, we expect to show increased antisaccade error rates in the 22q11DS group compared to typically developing controls and that poorer antisaccade performance will be associated with poorer performance on attention-dependent neuropsychological tasks (executive functioning) in both groups.

## Methods

The study protocol was approved by the University of Newcastle Australia Human Research Ethics Committee. Informed consent was obtained from all participants who took part in the study in accordance with the Code of Ethics of the World Medical Association (Declaration of Helsinki).

### Participants

We present the data from 25 adolescents (mean age 16.8 ± 2.9 years, 10 male subjects) with genetically confirmed 22q11DS recruited from the VCFS & 22q11 Foundation, Australia, and from local health services. The control subjects (HC) were 30 siblings of 22q11DS participants or other typically developing adolescents recruited from the community (mean age 16.5 ± 3.5 years, 14 male subjects). Exclusion criteria for the 22q11DS participants were the presence of the clinical phenotype of 22q11DS without a confirmed 22q11.2 deletion, a clinically detectable medical disorder known to affect brain structure (e.g. epilepsy or hypertension) or a history of head injury. Exclusion criteria for the HC sample were the presence of a genetic disorder or a major mental health problem. Additional exclusion criteria for both groups included a history of severe head injury, seizure disorder or other ocular, neurological or medical problems that could influence task performance. Visual acuity and auditory thresholds were assessed in all subjects prior to testing using a Snellen chart and audiometric assessment of hearing (range: 250 Hz–6 kHz). A psychiatrist (author: US) conducted a structured diagnostic interview [K-SADS; [29]] at the time of testing to determine 22q11DS participants’ diagnostic status. Eleven individuals with 22q11DS were identified as having (or having had) psychiatric diagnoses such as ADHD (*n* = 3), oppositional defiant disorder (*n* = 2), generalized anxiety disorder (*n* = 2), obsessive-compulsive disorder (*n* = 3), trichotillomania (*n* = 1) and major depressive disorder (*n* = 1). Six individuals were currently on medication; antipsychotics: risperidone (*n* = 2), mood stabilizers: valproate (*n* = 2), methylphenidate (*n* = 4) or SSRIs (*n* = 4). One participant was diagnosed with schizophrenia at the time of interview. The data of this participant was excluded because of non-compliance with task instructions for antisaccade and PPI.^a^

### IQ and executive functioning

#### Intellectual functioning

The Wechsler Abbreviated Scale of Intelligence [WASI 4 subscale version; [30]] was used to assess general intellectual functioning with verbal, performance and full-scale IQ calculated.

#### Planning task

A computerised planning task based on the Tower of London (ToL) task was designed. A goal and start configuration was shown simultaneously in the upper and lower half of the screen. The goal configuration was smaller in size and surrounded by a distinct square. An image of three pegs of decreasing heights and three balls of different colours were displayed. The task was displayed on a touch screen. Only one ball could be moved at a time. A ball could only be moved if there was no other ball on top, and three balls could be placed on the long peg, two on the medium and one on the shortest peg. The participants were instructed to transform the start state into the goal state by touching the appropriate ball (to activate it) and then touch the end-position to move the ball. The *planning task* ranged from 2–7 moves necessary for completion. There was no time limit to solve a problem. Variables recorded were moves above minimum, initial thinking time and subsequent thinking time. All participants received feedback at the end of each trial, consisting of a smiley face in different colours (yellow for perfect performance and purple for passing the trial) or a sad face when failing the trial. All participants also completed a motor control task, in order to control for motor movement time. This consisted of a five-move task that was broken down into 5 one-move tasks so that there should be no thinking time, and all time should be taken up by movement. This time was subtracted from the movement execution time. The participants completed the practice trials before commencing the task and followed by a two-move condition to understand the goal of the main task. These trials were not included in the data analysis.

### Experimental tasks

#### PPI measures

Electromyographic (EMG) recordings of sensorimotor gating of the acoustic startle eyeblink response were undertaken. Auditory stimuli were generated using a presentation software (Neurobehavioural Systems) and were presented binaurally through stereo headphones using a set-up similar to earlier published studies [31]. The startle stimuli were rectangular white noise (50-ms duration, 110 dB sound pressure level (SPL)). Pre-pulse stimuli consisted of two pure tones (high-pitch 1,400 Hz, 20 ms, 5-ms ramp or a low-pitch 800 Hz, 20 ms, 5-ms ramp) at 80 dB SPL (against ~70 dB SPL background noise) presented either 120 or 480 ms before startle stimulus onset. All acoustic stimuli were calibrated using a Bruel and Kaejer sound level meter (type 2231) and artificial ear (type 4151). There were three trial types: (i) startle baseline (startle probe only); (ii) 120-ms pre-pulse and (iii) 480-ms pre-pulse. There were several practice trials for each of the attended and unattended conditions and a total of ten instances in each condition (120 ms passive/active; 480 ms passive/active). The stimulus sequence commenced with the presentation of three startle baseline trials that were discarded and excluded from the analyses. This was followed by an alternating sequence of 20 startle baseline and 20 pre-pulse trials with a variable interstimulus interval of 10–12 s. Within this sequence, 120-ms and 480-ms pre-pulse trials were presented equally in a pseudorandomised order. Two conditions were presented, a passive and an active with the passive condition completed first by all participants. In the passive condition, participants were instructed to ignore the stimuli and make no overt response while watching a silent movie. For the active condition, participants were instructed to listen to the stimuli and were required to respond to the high-pitch tone by pressing a button as quickly and accurately as possible.

#### EMG recording and analyses

Bipolar silver/silver chloride electrodes were positioned above the orbicularis oculus muscle of the subject’s left eye to record the blink response (A/D rate of 1,000 Hz and amplified × 500). Impedances were reduced to less than 5 kΩ. EMG activity was band-pass filtered (1–200 Hz with 50 Hz notch). Epochs were extracted from 50-ms pre-startle to 300-ms post-startle stimulus onset, baseline corrected over the 50-ms pre-stimulus interval, rectified and averaged separately for each of the three trial types. Startle response amplitude was determined as the integral averaged under the curve occurring between 30 and 150 ms from the onset of startle stimulus. Eight 22q11DS participants did not complete the EMG session. Seventeen individuals with 22q11DS undertook PPI recordings (mean age 16.7 ± 2.8 years, 5 male subjects) and 19 HC (mean age 16.3 ± 4.0 years, 8 male subjects).

#### Antisaccade recording and analysis

Participants’ eye movements were recorded remotely using an Eyelink 1000 (1,000 Hz, SR Research, Ontario, Canada) linked to a host Dell Pentium IV PC processor and an auxiliary video display unit for observing the monitored eye. Each stimulus block was preceded by a calibration and validation procedure that required participants to fixate on a 3 × 3 matrix of centrally and peripherally located points on the computer screen. Each trial consisted of the following sequence: (i) a circular target was presented at the beginning of each trial; (ii) after 1,000 ms, the target was extinguished, a peripheral target was illuminated and a brief beeping signal was initiated and (iii) an extinction of the cue occurred after 3,000 ms or earlier if participant gaze was recorded within 1° (visual angle) of the target response area and antisaccade target had been initiated for greater than 1,000 ms. The target stimulus, a small red target of 0.9° in diameter, was presented on a black computer screen. The stimuli in each block were presented to the left or right of the screen in a balanced pseudorandom order. An antisaccade error occurred when the subject made a reflexive saccade towards the target before correctly making a saccade in the opposite direction. Antisaccade errors were identified using custom software and expressed as a percentage of the total. Seventeen individuals with 22q11DS undertook antisaccade recordings (mean age 17.1 ± 3.08 years, 7 male subjects) and 28 HC (mean age 16.7 ± 3.4, 13 male subjects).

### Analysis

SPSS 19 was employed for statistical analyses. In both participant groups, with the exception of antisaccade error and latency, the distribution of inhibition measures did not violate assumptions of normality (Shapiro–Wilks test) and parametric statistics were used in subsequent analyses.

PPI was determined as a percentage and calculated for each ISI in each stimulus condition using the formula: Percent PPI = 100 × [(startle pulse only units - pre-pulse-startle pulse response units)/startle pulse only units]. PPI measures were examined using repeated measures *t*-tests and mixed model ANOVA (pre-pulse interval [120 ms, 480 ms] × attention [passive, active] × group). Antisaccade accuracy, amplitude and latency (of correct and incorrect trials separately) were calculated using software designed for eye movement analysis, and antisaccade accuracy data were examined using one-way ANOVAs. Independent samples *t*-tests, chi square and one-way ANOVA were used to examine demographic and executive functioning differences between groups. Parametric correlational analyses were conducted to examine relationships between executive functions and performance measures on antisaccade and PPI. Threshold set for significance was 0.05.

## Results

A total of 55 adolescents were included in these analyses; however, not all participants completed both antisaccade and PPI components of the study. As such, sample size varied depending on the analysis. The groups (22q11DS/HC) did not differ from each other in age (*t*_(53)_ = 0.28, *p* = 0.78) or gender (*χ*^2^(1) = 0.25, *p* = 0.62), but IQ difference was greater than two standard deviations between groups (Full-scale IQ: mean (SD): 22q11DS = 75.4(15.3); HC = 104.4(15.8), Table 1).

**Table 1:** **Neuropsychological characteristics of the 22q11DS and HC groups**

Cognitive data	22q11DS (***n*** = 23)		HC group (***n*** = 27)		Statistic ***t***(df)
	Mean	SD	Mean	SD	
IQ					
Verbal IQ score	76.9	13.1	104.6	17.2	
Performance IQ score	72.9	14.0	108.0	14.7	
Full-scale IQ score	75.2	15.0	107.2	15.6	
Executive functioning Planning: Tower of London					
Moves above minimum	3.11	3.02	0.97	0.56	14.71 (1,48)**
Initial thinking time (ms) (3–5 moves)	5,572	1,992	11,148	7,670	11.46 (1,48)**
Subsequent thinking time (ms) (3–5 moves)	25,202	3,253	12,172	6,427	4.16 (1,48)*

*p < 0.05; **p < 0.001.

### Pre-pulse inhibition (PPI) task performance

Startle eyeblink responses were modulated by pre-pulse interval (120/480 ms: F(1,34) = 9.84, *p* = 0.004) and by condition (passive/active: F(1,34) = 35.98, *p* < 0.001). Both groups showed PPI to the 120-ms pre-pulse interval versus baseline startle response (no pre-pulse presented) in both the passive and active pre-pulse conditions (passive task: *t*(18) = 2.72, *p* = 0.014 HC subjects; *t*(16) = 5.35, *p* < 0.001 22q11DS subjects; active task: *t*(18) = 4.30, *p* < 0.001 HC subjects; *t*(16) = 2.58, *p* = 0.02 22q11DS subjects, Table 2).

**Table 2:** **Electromyographically recorded startle eyeblink responses at baseline (no pre-pulse presented) and following presentations of subtle acoustic pre-pulses at 120- and 480-ms lead intervals**

	22q11DS (***n*** = 17)		HC group (***n*** = 19)	
		%PPI or %PPF		%PPI or %PPF
Passive listening task				
Baseline startle	111.98 (±73.07) μV		133.2 (±117.3) μV	
120-ms pre-pulse	87.08 (±65.25) μV	22.8 (±5.6)	109.3 (±103.8) μV	17.1 (±8.3)
480-ms pre-pulse	136.08 (±81.6) μV	-24.4 (±11.7)	131.2 (±100.8) μV	-3.1 (±8.5)
Active discrimination task				
Baseline startle	94.9 (±69.1) μV		104.9 (±72.6) μV	
120-ms pre-pulse	73.7 (±61.0) μV	15.5 (±11.9)	68.6 (±53.1) μV	34.1 (±5.4)
480-ms pre-pulse	99.0 (±84.7) μV	-12.8 (±18.3)	114.2 (±86.2) μV	-23.3 (14.0)

Resulting percent change as pre-pulse inhibition (%PPI) or pre-pulse facilitation (%PPF) relative to baseline startle response (standard error mean in parenthesis).

**Figure 1:**
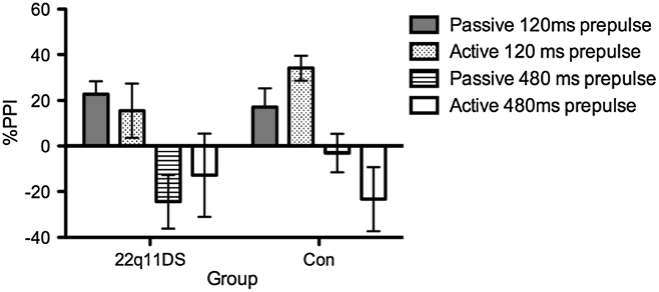
**Mean percent pre-pulse inhibition or pre-pulse facilitation for 120- and 480-ms pre-pulse lead intervals.** Mean percent pre-pulse inhibition (PPI) or pre-pulse facilitation (PPF) for 120- and 480-ms pre-pulse lead intervals relative to baseline startle response (no pre-pulse presented). Note differential attention effects on PPI and PPF between groups depending on performance on an auditory discrimination task on the pre-pulse (active) and not (passive).

The three-way interaction of pre-pulse interval by attention conditions by group approached significance (F(1,34) = 3.83, *p* = 0.059; Figure 1). *Post hoc* testing revealed a differential modulation of sensorimotor gating between groups. HC participants showed a significant increase in PPI in the 120-ms pre-pulse interval when directing attention to the pre-pulse, compared to the passive listening task (*t*(18) = -2.42, *p* = 0.026). In contrast, 22q11DS subjects showed a trend towards reduced PPI when directing attention to the pre-pulse (*t*(16) = -1.80, *p* = 0.09) at the 120-ms pre-pulse interval compared to the passive listening task. Moreover, 22q11DS subjects showed significantly greater PPF in the passive listening task at the 480-ms pre-pulse interval compared to the active listening task (*t*(16) = -2.31, *p* = 0.035). Figure 1 indicates both groups exhibit PPF; however, this differs between conditions, with the HC group exhibiting greater (although statistically non-significant) PPF during the active condition.

The ANOVA revealed no additional significant interactions with none of the following conditions reaching significance: interval by group (F(1,34) = 0.045, *p* > 0.05); condition by group (F(1,34) = 0.08, *p* > 0.05) nor interval by condition (F(1,34) = 0.00, *p* > 0.05).

### Antisaccade task performance

Group comparisons on antisaccade error, latency and amplitude showed the 22q11DS group made significantly more antisaccade errors compared to the HC group (F(1,43) = 28.93, *p* < 0.0001) but did not differ on measures of latency or amplitude (*p* > 0.05). These effects remained non-significant when separated by trial accuracy (correct/incorrect antisaccade). However, antisaccade error was influenced by response latency with increased latency for successful antisaccade inhibition trials in both groups (F(1,41) = 41.63, *p* < 0.0001) (Table 3).

**Table 3:** **Mean (SD) antisaccade parameters for 22q11DS and HC groups**

	22q11DS mean (SD) (***n*** = 17)	HC group mean (SD) (***n*** = 28)
Mean% error	51.3 (19.57)	23.7(16.36)***
Mean latency (ms)	257.99 (92.68)	261.24 (38.0)
Mean latency (ms) (correct)	300.45 (155.9)	279.95 (50.69)
Mean latency(ms) (incorrect)	221.49 (66.60)	204.55 (50.66)
Mean amplitude (^o^/s)	1.44 (0.12)	1.38 (0.07)

***p < 0.0001.

### Associations amongst antisaccade, PPI and executive functioning

Statistical significance of the difference between correlation coefficients for the 22q11DS and HC groups was examined because the patterns of association differed between the groups on several measures. Where the groups did not differ significantly (*z*_(obs)_: -1.96–1.96), we performed partial correlations controlling for group membership to explore the relationship between the key variables of interest. Bonferroni-adjusted significance level of 0.004 was calculated to account for the increased possibility of type-I error. Following Bonferroni correction, antisaccade error was moderately associated with executive function measures of planning ability, with increased moves on the Tower of London task associated with increased rates of antisaccade error (df = 37, *r* = 0.447, *p* = 0.004). Zero-order correlations indicated a moderate effect of group (*r* = 0.630).

## Discussion

This study set out to characterise inhibitory information processing in 22q11DS, using multiple measures (active/passive SEM (PPI and PPF); antisaccade inhibition) and examine potential associations with executive functioning. This is the first study to systematically examine automatic and controlled components of information processing and their relationship to executive functioning in 22q11DS. We report evidence of impaired controlled attentional processes, i.e. antisaccade inhibition in 22q11DS individuals, as well as some evidence (though statistically non-significant) of sensorimotor gating deficits—in a prepulse inhibition paradigm with reduced attentional modulation of the startle eyeblink response.

Previous studies of PPI in 22q11DS have found impaired sensorimotor gating compared to typically developing controls when employing a passive PPI task [14]; however, group differences on the passive PPI component of the task were not reported in the present study. Differing methodologies including lead intervals, levels of white background noise, relative stimulus intensities and number of trials presented may account for this disparity [18, 32]. Additionally, the effects of age on PPI may explain the difference in results, with a younger sample recruited by Sobin and colleagues [14]. Others have recently reported that levels of PPI increase with age [33], indicating that the age of our cohort may have contributed to the differences between this study and previous reports of PPI in 22q11DS [18, 17]. We are currently limited to speculation regarding the maturational trajectory of PPI in clinical groups; however, based on the current findings, differences in a PPI paradigm may best explain the low levels of PPI reported for both 22q11DS and HC groups in this study compared to previous reports in both 22q11DS [14] and other populations which typically report PPI in healthy control participants around 50%–60% [18]. These floor effects in our passive PPI condition may explain our failure to replicate associations between measures of executive functioning and PPI that have been reported previously in 22q11DS [13] and clinical conditions prevalent in 22q11DS [autism spectrum disorder; ASD [34]].

The results of the present study indicate attention-modulated disruption of sensorimotor gating in 22q11DS. One of the purposes of this study was to investigate higher- *and* lower-order levels of information processing in 22q11DS. Therefore, we incorporated a PPI paradigm that systematically examined relatively automatic (passive task) and controlled (active task) attention. During active attendance, the effects of an acoustic pre-pulse are typically magnified, with an increase in startle suppression (i.e. increase in PPI) at short lead intervals and increased startle (i.e. decrease PPI or increase PPF) at longer lead intervals [17]. We showed that individuals with 22q11DS appear to exhibit appropriate inhibition and facilitation of the startle response during the passive task, indicating normal early or automatic information processing. However, by failing to display the normal pattern of greater PPI and PPF during attended, rather than ignored, pre-pulses, the 22q11DS group exhibited a dysfunction in controlled attentional processing, rather than a pre-attentive dysfunction.

### Antisaccade inhibition

This is the first time antisaccade inhibition has been examined in 22q11DS. Our results were consistent with reports from other clinical populations [schizophrenia [25], first episode psychosis [35]] indicating that 22q11DS participants were less able to inhibit an automatic pre-potent response (i.e. increased antisaccade error). Increased antisaccade error in 22q11DS may reflect the inhibitory demands of the task and support our PPI findings of impaired controlled attention processes in 22q11DS. Our findings are also consistent with studies that show poor antisaccade performance in ultra-high risk for schizophrenia [36] and first episode schizophrenia groups [37].

Our passive PPI task was uncorrelated with the antisaccade task. This is consistent with previous studies in schizophrenia that show no association between passive PPI and antisaccade accuracy [38]. We partly concur with Swerdlow et al. [39] that the absence of association between passive PPI and antisaccade indicates that the measures are partly "dissociable and non-redundant" (p336) measures of information processing, resembling tasks tapping early versus late portions of the information processing chain. The present study shows poorer antisaccade accuracy, as well as attention-modulation deficits in PPI in 22q11DS. Taken together, these findings suggest a more cortical, in particular prefrontal, than subcortical (i.e. superior collicular) dysfunction in our 22q11DS sample.

### Limitations

Several limitations are associated with this study. The lack of an intellectually matched control group and convenience recruitment approach to participant selection is inherent to research of this nature and a limitation in 22q11DS research noted by our group previously [40]. In addition, 22q11DS is associated with a range of mental health conditions (see Participants); and these diagnoses and the medications used to treat them may have influenced our findings. However, these clinical diagnoses also reflect a defining characteristic of 22q11DS and were too few in number and varied in type to control statistically. Regarding medication, it is unclear how best to control for the influence of medication status given the medication participants reported using at the time of testing may have improved or impaired performance on inhibition tasks. In the future, our findings may be strengthened with additional controls for medication and clinical status in a larger sample.

### Clinical implications

The path of cognitive maturation in 22q11DS remains unclear; and future studies may benefit from the inclusion of participants aged across the lifespan to help determine whether inhibition processes are underdeveloped, delayed or constant across the lifespan in this group. Future studies may also benefit from exploring the influences of GABA, dopamine and glutamate transmission [13], as they are likely to be involved in the disturbed attention-modulated PPI and, we would suggest, the poor antisaccade performance reported in the current sample.

## Conclusions

The present study undertook a comprehensive examination of inhibitory functioning in 22q11DS using multiple measures and examined their relationship to executive functioning. While impaired PPI was not reported using the current paradigm, we identified similarities with findings reported in clinical populations associated with 22q11DS such as schizophrenia that may indicate shared dysfunction of inhibition pathways in these groups.

## Endnote

^a^The pattern of associations reported for the antisaccade task did not differ when this participant was included in analysis and we did not have PPI data for this participant.

## Abbreviations

22q11DS: 22q11.2 deletion syndrome
EMG: Electromyographic
ERP: Event-related potential
PPF: Pre-pulse facilitation
PPI: Pre-pulse inhibition
SEM: Startle eyeblink modification.
